# Effect of Keishibukuryogan, a Japanese Traditional Kampo Prescription, on Improvement of Microcirculation and Oketsu and Induction of Endothelial Nitric Oxide: A Live Imaging Study

**DOI:** 10.1155/2017/3620130

**Published:** 2017-07-13

**Authors:** Tsutomu Tomita, Aki Hirayama, Hirofumi Matsui, Kazumasa Aoyagi

**Affiliations:** ^1^Timelapse Vision Inc., 5-23-11 Honcho, Shiki, Saitama 353000, Japan; ^2^Department of Materials Science, Graduate School of Pure and Applied Sciences, University of Tsukuba, 1-1-1 Tennodai, Tsukuba, Ibaraki 3058573, Japan; ^3^Center for Integrative Medicine, Tsukuba University of Technology, 4-12-7 Kasuga, Tsukuba, Ibaraki 3058521, Japan; ^4^Faculty of Medicine, University of Tsukuba, 1-1-1 Tennodai, Tsukuba, Ibaraki 3058575, Japan

## Abstract

Oketsu is a characteristic condition that plays an important role in Kampo, Japanese traditional medicine, and includes multiple aspects of hemodynamic disorders. This study aims to clarify the microcirculation of Oketsu and the pharmacological effect of Keishibukuryogan, an anti-Oketsu Kampo prescription, using live imaging techniques. Oral administration of Keishibukuryogan induced significant vasodilation of murine subcutaneous arterioles compared to the preadministration level. This vasodilatation peaked 60 min after administration and persisted for 90 min. The blood velocity in the subcutaneous capillary was also increased by Keishibukuryogan in generally the same manner. In rat mesenteric arterioles, Keishibukuryogan administration improved microhemodynamic parameters, including the resolution of erythrocyte congestion and the cell-free layer, which are representative of Oketsu pathology. Live imaging revealed an increase of diaminofluorescein-2 diacetate fluorescence, a nitric oxide (NO) specific reagent, in the arterial endothelium following Keishibukuryogan administration. This fluorescence was most remarkable at vascular bifurcations but was present throughout the mesenteric arterioles. This study demonstrates the successful imaging of Oketsu pathology with respect to microcirculation and the anti-Oketsu effects of Keishibukuryogan, namely, vasodilation of arterioles, increased blood velocity, and resolution of erythrocyte congestion. The anti-Oketsu effect of Keishibukuryogan is related to endothelial NO production.

## 1. Introduction

Oketsu is a characteristic pathophysiology in Kampo, Japanese traditional medicine, and oriental medicines, a concept absent from western medicine. Oketsu is a condition characterized by a disturbance in the flow of “Ketsu,” which means blood and total body fluid. Although the direct translation of Oketsu to medical terminology is ischemia, Oketsu represents a broader pathology status, which includes oversensitivity to cold, flushes, back pain, menstrual disorder, hemorrhoids, and sleeping disorders. The circulation disorder aspect of Oketsu is not restricted to the arterial and venous systems but extends to capillaries. Because this pathophysiological concept is unique to oriental medicine, Kampo prescriptions are widely used as therapeutic strategies for Oketsu. However, much of the pathophysiology of Oketsu and the pharmacological effects of anti-Oketsu drugs remain unclear.

Blood circulation in the arterial and venous systems has been analyzed in detail from the level of the aorta to small vessels using a progression of techniques from Doppler ultrasonography to magnetic resonance imaging. On the other hand, technological difficulties are encountered in visualizing the hemodynamics of the capillary system, which is the key site of Oketsu pathology. Moreover, it is known that nitric oxide (NO) and reactive oxygen species (ROS) are closely involved in microcirculation kinetics, and oxidative stress plays a large role in the associated disorders [1]. However, analysis of NO kinetics at a microcirculation level also entails technical challenges.

In this study, we aimed to investigate the pharmacological effect of Keishibukuryogan, a major anti-Oketsu prescription, on microcirculation using cutting-edge live imaging techniques. Specifically, live imaging of the effects of Keishibukuryogan on subcutaneous vessels was performed using a murine model. Further, we also evaluated NO production in rat mesenteric arteries following Keishibukuryogan administration using live imaging.

## 2. Materials and Methods

### 2.1. Materials

C57B/6 mice and Wistar rats were purchased from Japan SLC (Hamamatsu, Japan). N^G^-Monomethyl-L-arginine acetate (L-NMMA), diaminofluorescein-2 diacetate (DAF-2 DA), and urethane were obtained from Dojindo Molecular Technologies, Inc. (Kumamoto, Japan), Goryo Chemical (Sapporo, Japan), and Sigma-Aldrich Japan (Tokyo, Japan), respectively. Confocal laser scanning microscope images were obtained using an LSM 700 manufactured by Zeiss (Oberkochen, Germany) and analyzed with ImageJ ver.1.45s image analysis software (NIH, USA). Keishibukuryogan dry extract granules were provided by Tsumura Co., Ltd. (Tokyo, Japan). The Keishibukuryogan extract used in this study contained Cinnamomi Cortex, Paeoniae Radix, Semen Persicae, Poria Sclerotium, and Moutan Cortex. The daily dose of Keishibukuryogan extract (7.5 g per day) contained 3 g of each of the above ingredients. Live imaging experiments were carried out at the Timelapse Vision Inc. under the approval of the local ethics committee.

### 2.2. Live Imaging of Murine Subcutaneous Vessels

Male C57B/6 mice (8 weeks old) were used in experiments (*n* = 3). The experiments were conducted under urethane (1.5 g/kg, i.s.) anesthesia. Following an incision, the ventral side skin of the mouse was peeled off, and the subcutaneous blood vessel was positioned on a glass plate. The vessel was covered with a thin vinylidene chloride film and fixed to prevent moisture evaporation. Keishibukuryogan was suspended in hot distilled water at 100 mg/mL and cooled to room temperature. A dose of 300 mg/kg was transesophageally administered to the mice using a gastric tube.

For live imaging, microcirculation of the subcutaneous blood vessels was recorded using real-time imaging from before Keishibukuryogan administration and every following 30 min up to a total of 180 min. The inner vessel diameter and blood velocities were analyzed from the obtained images with ImageJ ver.1.45s software. The changes in diameter and velocities were evaluated in both arterioles (10 to 20 *μ*m) and capillaries (<10 *μ*m). The inner diameter of vessels and blood vessel velocities were analyzed at 0, 30, 60, 90, 120, 150, and 180 min. The diameter of blood vessels was measured in triplicate at three points, for a total of nine measurements for each animal, on the screens. The blood flow velocity was calculated based on measurements of erythrocyte locations within the capillaries in individual images. The distance travelled by individual erythrocytes was divided by the elapsed time to provide the blood flow velocity (*μ*m/sec). Blood flow velocity was determined by performing quadruplicate measurements at a single point and reported as average values. In addition, we measured the heart rate of animals for evaluation of hemodynamics.

### 2.3. Live Imaging of Microcirculation and Nitric Oxide Release in Rat Mesenteric Arteries

Female Wistar rats (9 weeks old) were used (*n* = 3 for each group), and experiments were conducted under urethane (1.5 g/kg) anesthesia following overnight fasting. After an approx. 2 cm incision was made in the peritoneum, the ileum was drawn out and the mesentery was spread on a glass plate, which was covered by a thin vinylidene chloride film to prevent moisture evaporation. The imaging site was determined using an intravital microscope, and a catheter was inserted into a vessel upstream of the set site to obtain a perfusion route. Live imaging was performed in the same manner as for the murine subcutaneous blood vessels. As shown in Figure 4(a), sites reflecting upstream, middle, and peripheral sections of the mesenteric artery were established in each rat. The effects of Keishibukuryogan administration were investigated using the following three groups: the control group given DAF-2DA only, the Keishibukuryogan group given DAF-2DA and Keishibukuryogan, and the L-NMMA group given DAF-2DA and L-NMMA. For the Keishibukuryogan group, 300 mg/kg of a 100 mg/mL solution of Keishibukuryogan was administered to the rats using transesophageal gastrotubing.

NO production was investigated using the NO-specific fluorescent indicator DAF-2 (the optimum excitation wavelength is 495 nm and fluorescence wavelength is 515 nm). After live imaging of microcirculation, DAF-2DA (50 *μ*M) was administered through the route and perfused locally for 3 min. Immediately thereafter, a baseline fluorescence image was obtained using confocal laser scanning microscopy which was set with the excitation wavelength at 488 nm by using argon laser and the fluorescence was detected through a 492 nm long pass filter. In the Keishibukuryogan group, after confirming the disappearance of fluorescence, Keishibukuryogan was administered intragastrically 30 min later. Then, after another 30 min, DAF-2 DA was perfused again and a second round of images were obtained at the same site as the initial imaging, and the change in fluorescence intensity of DAF-2 before and after administration of Keishibukuryogan was analyzed. In each group, images were acquired in a total of 6 to 9 regions from 3 rats. In the control group, the second fluorescence images were obtained 60 min after the initial baseline imaging, without any treatment between the first and second perfusion of DAF-2DA. The other process was performed in the same manner as for the Keishibukuryogan group. In the L-NMMA group, 25 mg/kg of the NO synthase inhibitor L-NMMA was intravascularly administered 5 min before the second DAF-2DA perfusion, and a second dose of L-NMMA was added to the DAF-2DA solution at a final concentration of 2.5 mg/mL. Imaging was performed in the same manner as for the control group. For the analysis, regions of interest were established at the site of observation, and the average fluorescence intensity in that area was obtained using ImageJ software.

### 2.4. Statistical Analysis

Statistical analysis was performed using Prism 6 for Mac OS X (Graphpad Software Inc., La Jolla, CA, USA) with a multiple comparison test using the Holm-Sidak method using one-way ANOVA with repeated measurement.

## 3. Results

### 3.1. Keishibukuryogan Induced Vasodilation and Improved Blood Flow Velocity in Murine Subcutaneous Vessels

**Figure 1:**
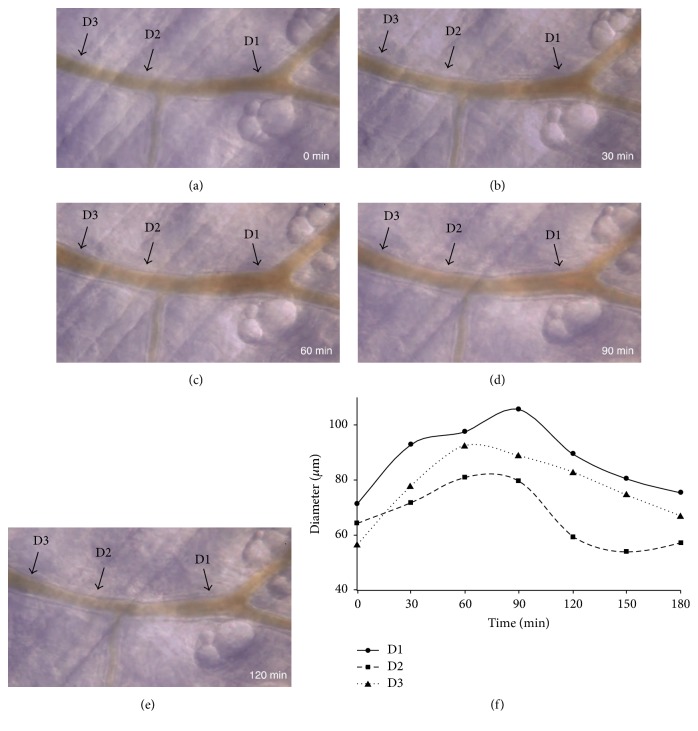
Live imaging of murine subcutaneous vessels before and after the administration of Keishibukuryogan. Representative images are shown. (a) shows the image before Keishibukuryogan administration, while (b)–(e) show images of the same site at 30, 60, 90, and 120 min after administration. (f) shows the change in vascular diameter at sites D1–D3 shown in (a)–(e). Maximum vasodilation from 60 to 90 min after Keishibukuryogan administration was confirmed. The vascular diameter was restored to preadministration levels at approx. 180 min.

**Figure 2:**
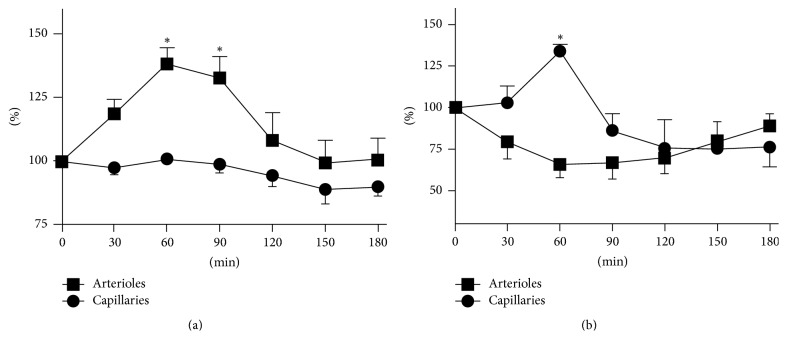
Changes in vasodiameter (a) and blood flow velocity (b) of murine subcutaneous arterioles (■) and capillaries (●) before and after administration of Keishibukuryogan. Both (a) and (b) are expressed as a percentage, with preadministration expressed as 100%. ^*∗*^*p* < 0.05.

Representative images of live imaging of subcutaneous blood vessels in mice are shown in Figures 1(a)–1(e), and changes in vessel diameter at chosen sites are shown in Figure 1(f). Total changes in vessel diameter of 3 different mice following Keishibukuryogan administration are shown in Figure 2(a), and changes in blood flow velocity are shown in Figure 2(b). Significant dilation of the vessels peaked after 60 min and effects lasted for a total of 90 min on the arterioles (*p* < 0.05). No significant changes were observed in the capillaries.

Blood flow velocities were evaluated in the vessels with the diameter of arterioles (10 to 20 *μ*m) and capillaries (<10 *μ*m). The velocities varied considerably depending on the site and were 701 ± 257 and 298 ± 149 *μ*m/sec, respectively (mean ± SE), immediately prior to Keishibukuryogan administration. A significant increase in blood flow velocity was observed at 60 min after administration in the capillaries (*p* < 0.05), but this decreased after 90 min. Before the administration of Keishibukuryogan, the heart rate of the mice was 651 ± 11 bpm. The heart rates at 30, 60, 90, 120, 150, and 180 min after the administration were 683 ± 8, 673 ± 4, 679 ± 9, 670 ± 12, 674 ± 6, and 676 ± 5% (mean ± SE), and no significant differences were observed.

### 3.2. Keishibukuryogan Improved Oketsu Accompanied by Nitric Oxide (NO) Increase in Rat Mesenteric Arteries

**Figure 3:**
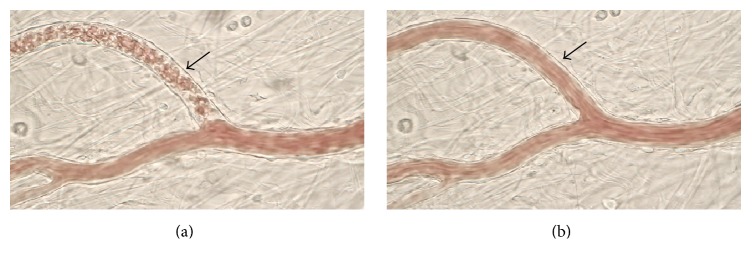
Live imaging of rat mesenteric arterioles before (a) and 60 min after (b) the administration of Keishibukuryogan. Representative images are shown. Prior to Keishibukuryogan administration, erythrocyte congestion and cell-free plasma layer (plasma pocket) as indicators of Oketsu status (shown by arrows) were observed. At 60 min after administration, these Oketsu symptoms were resolved.

**Figure 4:**
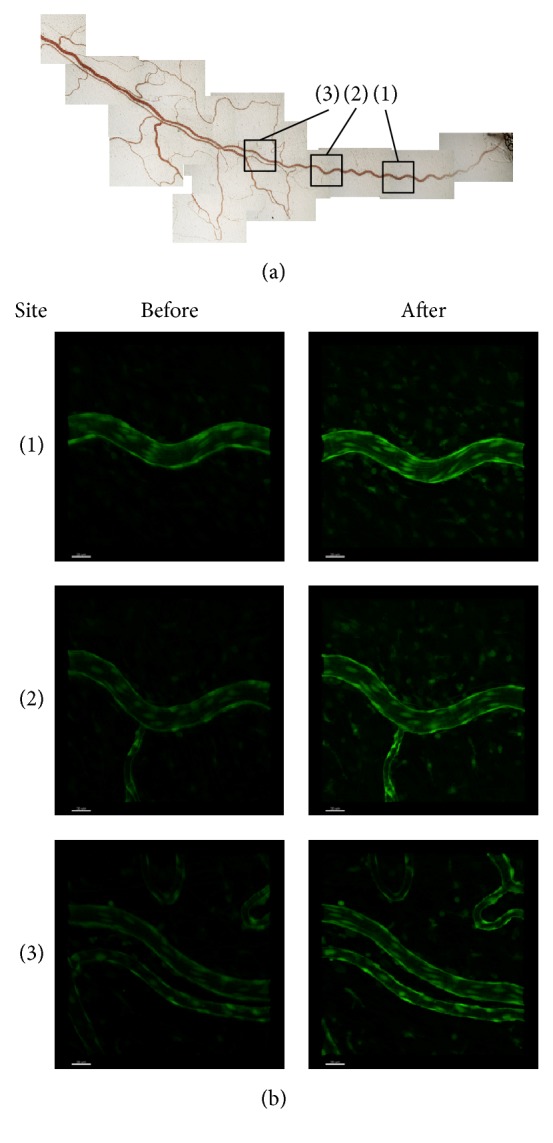
Imaging of the mesenteric arterioles before and 60 min after the administration of Keishibukuryogan in rats. Representative images are shown. (a) indicates the site of the mesentery used for imaging and (b) shows the DAF-2 fluorescence images of the corresponding sites. The images of (a) (1)–(3) and (b) (1)–(3) correspond to the same site. Increases in NO levels were observed in all sites following Keishibukuryogan administration, particularly in endothelial cells.

**Figure 5:**
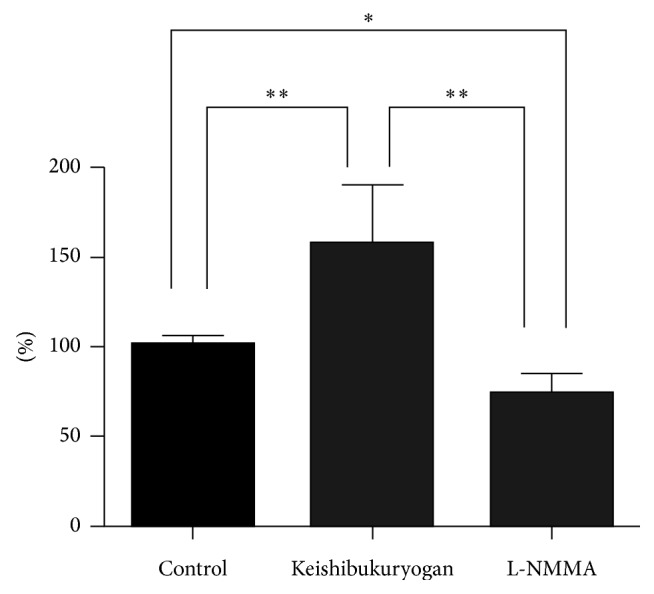
Changes in NO levels following the administration of Keishibukuryogan. DAF-2 fluorescence intensity was significantly increased in the Keishibukuryogan group compared with the control group. Also, DAF-2 fluorescence intensity was significantly decreased in the L-NMMA group. ^*∗*^*p* < 0.05, ^*∗∗*^*p* < 0.01.

Representative images of live imaging of the rat mesenteric arterioles are shown in Figure 3. Meanwhile, typical fluorescence images of rat mesenteric arterioles are shown in Figure 4, and changes in fluorescence intensity are shown in Figure 5.

In rat mesenteric arterioles before Keishibukuryogan administration, erythrocyte congestion in the capillaries and broadening of the cell-free layer, in which the plasma layer lacks erythrocytes, the so-called plasma pocket phenomenon, were observed (Figure 3). These circulatory deficiencies corresponded to aspects of Oketsu. Further, about 60 min after Keishibukuryogan administration, the circulatory deficiencies disappeared, and consistent blood flow, as observed in other regions, was confirmed.

In fluorescence imaging, fluorescence was observed in arteriolar vascular endothelial cells in the control group even in an unstimulated state, reflecting constitutive NO production in the endothelium (Figure 4(b)). The increase of fluorescence in the endothelium with the second DAF-2DA administration was 101.0 ± 2.4% as compared to the first imaging in the control group, which did not indicate a significant change (Figure 5). On the other hand, in the Keishibukuryogan group, a significant increase in DAF-2 fluorescence was observed after administration (157.7 ± 10.9%, *p* < 0.01). This increase in fluorescence was widely observed from upstream to the periphery of the mesenteric artery, while remarkable changes were observed at bifurcations, as shown in Figure 4(b). On the other hand, in the L-NMMA group, DAF-2 fluorescence intensity significantly decreased (74.2 ± 3.6%, *p* < 0.05) as compared with the control group.

## 4. Discussion

Oketsu is a condition that is particularly emphasized in Kampo and oriental medicine and is considered to consist of erythrocyte factors including membrane charge and deformability, blood viscosity, thrombocytic agglutinability, and endothelial function [2]. In this study, we aimed to clarify the blood circulation and NO dynamics in Oketsu pathology using live imaging techniques.

A murine model was employed for the subcutaneous blood vessel experiment and a rat model was used for investigation of NO dynamics in our study. Because murine subcutaneous vessels are composed of a series of vasculatures from large arteries to capillaries, they are suitable for measuring the change in blood vessel diameter due to contraction and relaxation. On the other hand, the diameters of rat mesenteric vessels are varied, and the reactivity is relatively poor. Moreover, our method of detecting NO by intravascular perfusion of DAF-2DA required cannulation on a vessel upstream of the observation area, and this process raised a technical limitation depending on the size of animals.

There are currently no established animal models of Oketsu pathophysiology. In this study, we examined conditions that are similar to Oketsu, by placing the exposed abdominal lateral skin on a fixing glass plate for the murine subcutaneous vessel model and by extracting the ileum from the abdomen and placing it on a fixing glass plate for the rat mesenteric arteriole investigation. As a result, erythrocyte congestion in the arterioles and capillaries and expansion of the cell-free layer were observed. While we cannot assume that these conditions mimic the pathology of Oketsu exactly, at a minimum, both erythrocyte and vascular factors, that is, erythrocyte congestion and vascular contraction, were reproduced. As a model of peripheral congestion in human small arteries, but not Oketsu, Ong and colleagues reported a series of studies using arterioles within the rat cremaster muscle, which resembles the rat mesentery in this study [3]. Particularly, generation of the plasma pocket phenomenon, involving the expansion of the cell-free layer, was demonstrated in this study and in microcirculation studies in general; moreover, the association with NO and ROS kinetics has been previously reported [4–6]. Because the situation of Oketsu reflects both erythrocyte and vascular factors, the existence of this cell-free plasma layer may be closely related to Oketsu pathology. Our results showed the anti-Oketsu effects of Keishibukuryogan, via its effect on vasodilation, improvement of blood flow velocity, and reduction of erythrocyte congestion and the cell-free layer, which are in agreement with the concept of Oketsu.

In this study, as the arterioles expanded, blood flow velocity at the same site decreased whereas in the capillary vessels blood flow velocity simultaneously increased (Figures 2(a) and 2(b)). Since the change of capillary diameter is much smaller than that in the arteriole, the increase in capillary blood flow indicates that the blood volume at the observation site increased. Even in the arteriole, the actual measured value of the blood flow velocity at 60 min after the administration is 66% of the velocity before the administration and it is higher than the predicted value (51%) calculated from the blood vessel diameter on the assumption that the blood flow rate does not change. Thus, the blood flow volume in the arteriole also increased. Consequently, Keishibukuryogan increased the blood flow volume with the increase in blood vessel diameter and blood flow velocity as its anti-Oketsu effect.

NO is considered to be an endothelium-derived relaxing factor and is thus regarded as a physiologically active substance of extreme importance in circulatory regulation [1]. Although NO is expected to be intimately involved in Oketsu, few studies have investigated this relationship, especially in vivo [7–9]. We previously reported that Kangenkaryu, an anti-Oketsu reagent, increased NO metabolites and affected the NO-superoxide balance in humans [10]. A series of studies from Ng and colleagues revealed the close relation between the formation of asymmetric cell-free layer widths and its influence on the uneven distribution of NO-superoxide balance in the downstream flow of an arteriolar bifurcation [5, 6]. These reports suggested the close relationship between Oketsu and NO and ROS dynamics. Meanwhile, Keishibukuryogan is a representative anti-Oketsu drug in Kampo. The in vitro relationship between its main pharmacological anti-Oketsu effect and NO has been reported, in which Keishibukuryogan showed inhibitory effects on inflammatory cytokines in skin endothelial cells [11]. In regard to the relationship with oxidative stress, the suppression of lipid peroxidation on erythrocyte membranes induced by 2,2-azo-bis(2-amidinopropane) dihydrochloride (AAPH) and the suppression of neuronal cell death induced by sodium nitroprusside have been investigated [12, 13]. Moreover, regarding the components of Keishibukuryogan, cinnamic aldehyde contained in cinnamon, paeoniflorin contained in* Paeonia lactiflora*, and amygdalin contained in Semen Persicae are known to increase peripheral blood flow [14–16], and cinnamic aldehyde and paeoniflorin reportedly showed anti-inflammatory effects involving NO-related pathways [15, 17–20]. These results strongly suggest that the anti-Oketsu effect of Keishibukuryogan is closely related to NO and ROS; however, most of these reports involved in vitro studies, with few reports of in vivo effects [21].

Our imaging study of rat mesenteric arterioles clearly imaged the improvement of Oketsu status following Keishibukuryogan administration, evidenced by the increase in DAF-2 fluorescence. Since DAF-2 has high NO specificity [22] and L-NMMA treatment decreased the fluorescence intensity, this indicates that the anti-Oketsu effect of Keishibukuryogan can be attributed to a NO-related mechanism. An increase in NO production was observed mainly in the endothelium 30 min after Keishibukuryogan administration, suggesting that eNOS was the target enzyme of Keishibukuryogan. This finding is in agreement with a previous in vivo study showing effects of Keishibukuryogan on both eNOS and iNOS in the aorta of an adjuvant-induced arthritis model [21]. On the other hand, the vasodilation effect of Keishibukuryogan persisted for a total of 120 min after administration, whereas the increased blood flow was maintained for only up to 60 min, subsequently decreasing despite the increases in vessel diameter. This suggests that Keishibukuryogan has rapid pharmacological effects in erythrocytes themselves; however, further studies are warranted to clarify this point.

This study is the first live imaging of Oketsu pathology and the anti-Oketsu pharmacological effects of Keishibukuryogan. Additionally, we revealed the relationship between NO and Oketsu and Keishibukuryogan effects using live fluorescence imaging. Because Oketsu is a traditional and complicated concept, its translation to modern scientific terminology has been challenging. While there continue to be a number of technical limitations, our approach represents a promising method to clarify the concept of Kampo pathophysiology as well as the pharmacology of Kampo prescriptions.
